# DV21 decreases excitability of cortical pyramidal neurons and acts in epilepsy

**DOI:** 10.1038/s41598-017-01734-z

**Published:** 2017-05-10

**Authors:** Min Xu, Peng Sun, Ying Zhang, Ci-Hang Yang, Xin Wei, Xiao-Xia Ma, Chong-Ren Yang, Kun-Ming Ni, Ying-Jun Zhang, Xiao-Ming Li

**Affiliations:** 1 Department of Neurobiology, Institute of Neuroscience, Key Laboratory of Medical Neurobiology of the Ministry of Health of China, Joint Institute for Genetics and Genome Medicine between Zhejiang University and University of Toronto, Collaborative Innovation Center for Brain Science, Zhejiang University School of Medicine, Hangzhou, Zhejiang China; 2 State Key Laboratory of Phytochemistry and Plant Resources in West China, Kunming Institute of Botany, Chinese Academy of Science, Kunming, China; 3 Center for Pharmaceutical Sciences, Faculty of Life Science and Technology, Kunming University of Technology and Science, Kunming, China

## Abstract

Epilepsy is one of the most common neurological disorders and the administration of antiepileptic drugs (AEDs) is the most common treatment. Although there are more than 15 AEDs available, a third of epilepsy patients remain refractory to available drugs, so novel effective drugs are needed. Here, we found that DV21, which is a natural triterpenoid compound extracted from plants of the Asclepiadaceae family, significantly decreased the incidence and stages of seizures in three classical drug-induced acute seizure models in C57BL/6 mice. Furthermore, we also found that the antiepileptic effect of DV21 might be partly mediated through reducing the excitability of cortical pyramidal neurons by increasing M current, which are low-threshold non-inactivating voltage-gated potassium currents. Moreover, the application of XE991, an inhibitor of M current, could block most the antiepileptic effect of DV21. Taken together, our results indicated that DV21 might be a novel leading compound for the treatment of epilepsy.

## Introduction

Epilepsy is one of the most common disabling neuropsychiatric disorders characterized by recurring and unprovoked seizures, and affected individuals often require lifelong medication^1^. More than approximately 65 million people worldwide have been affected, and 150,000 new cases are diagnosed every year in the United Stated alone^2^. Currently, the most common treatment for epilepsy is the administration of antiepileptic drugs (AEDs), and different types of seizures are treated with different medications. Despite the current availability of more than 15 AEDs, a third of patients remain refractory to available drugs^3^. As a consequence, it is necessary and urgent to find newer and more effective drugs.

Traditional Chinese Medicine (TCM) and ethnopharmacology have been important resources of novel active natural products that could serve as leads and scaffolds for developing into efficacious drugs that are needed^4^. In southwestern China, some plants of the Asclepiadaceae family have been used to treat epilepsy by local residents^5^. We identified an activated small natural molecule, termed DV21 here (**Spatial Structure** Supplementary Fig. 1a). DV21, a natural triterpenoid compound, can be found in various natural sources, especially in olive oil^6^. The compound has attracted much interest due to its proven pharmacologic safety and many biologic activities^7, 8^, such as its anti-inflammatory, antiviral, antidiabetogenic, and antitumoral and antiastrocytomal properties.

Here, we investigated the effects of DV21 in three different classical drug-induced acute epilepsy models, which have different mechanisms and mimic different types of clinical seizures in C57BL/6 mice. The results indicated that DV21 could significantly delay the onset of seizures and decrease the incidence and stages in all epilepsy mice models. Furthermore, we found that the antiepileptic effect of DV21 might be partly mediated by reducing the excitability of cortical pyramidal neurons by increasing M current, which are slow, low-voltage-activating, non-inactivating K^+^ currents^9^ and are thought to play a pivotal role in mediating excitability control and early spike-frequency adaptation in neurons^10^. Furthermore, the application of XE991, which is an inhibitor of M current, could block most the antiepileptic effect of DV21 both *in vivo* and *in vitro*. Our findings suggest that DV21 may be a new potential antiepileptic drug.

## Results

### Screening and testing the antiepileptic activity of DV21 on zebrafish seizure models

We obtained DV21 from the plants of the Asclepiadaceae family (*Dregea volubilis* (L. f.) Benth. ex Hook. f.) by high-throughput screening, which showed antiepileptic effects in zebrafish pentylenetetrazole (PTZ)-induced seizure models. By increasing concentrations of DV21, increased antiepileptic activity was observed, which could more strongly suppress the movement of the seizure model zebrafish (Supplementary Fig. 1b). Furthermore, we compared the antiepileptic effects of different concentrations of DV21 with those produced by several other clinical drugs in the same models (Supplementary Fig. 1c). The results showed that increasing concentrations of DV21 induced stronger antiepileptic effects, and accordingly, we concluded that the median effective dose of DV21 is 3.5 µM. (Supplementary Fig. 1d).

### DV21 decreases seizure incidence and severity in a PTZ-induced mouse model

**Figure 1:**
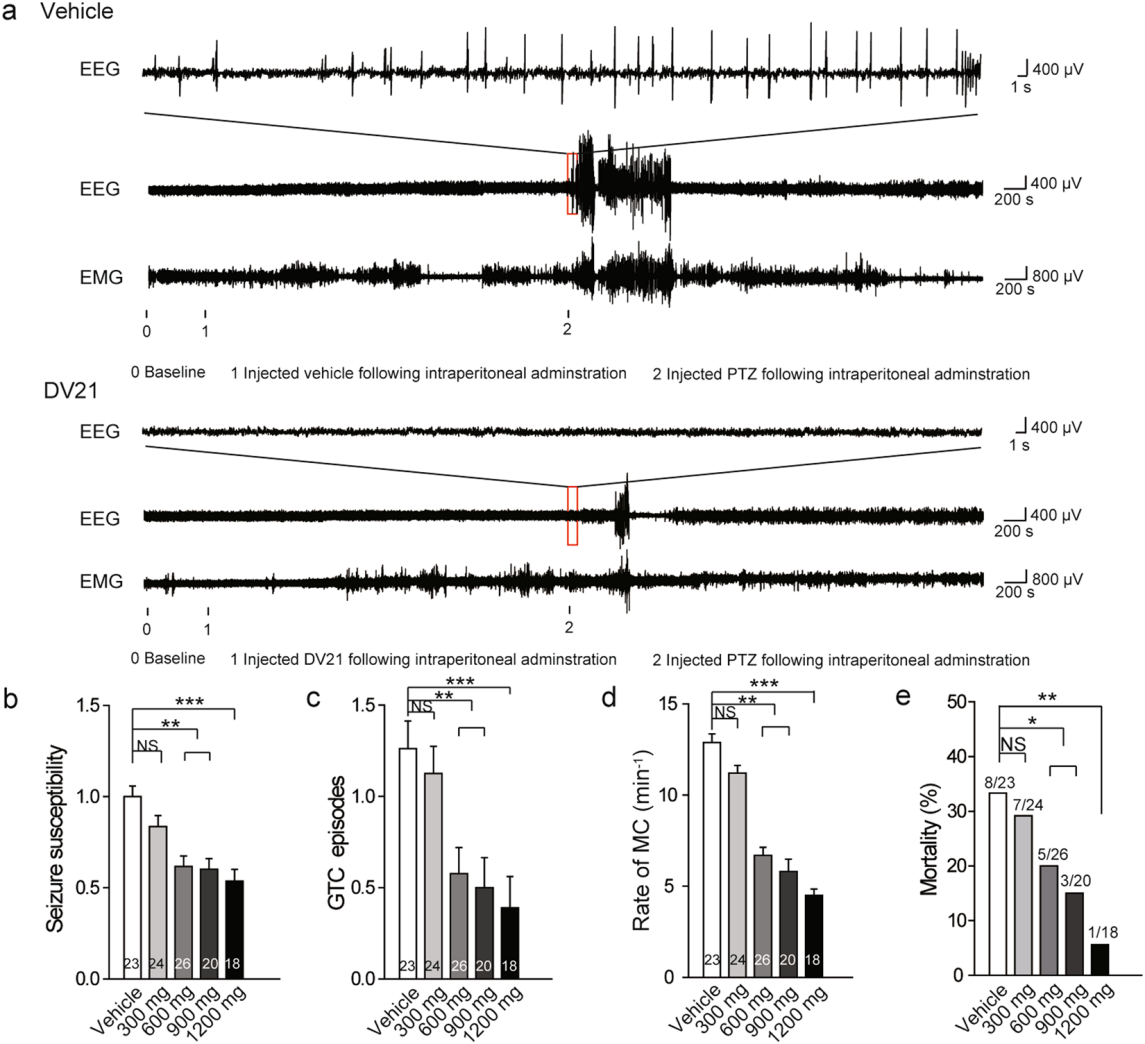
DV21 decreases PTZ-induced seizure incidence and severity. (**a**) Representative compressed electroencephalogram and electromyogram from a cortical lead depicting an electrographic seizure in a C57BL/6 mouse with seizure behaviors identified at the time of occurrence. Background EEG baseline is shown before seizure onset. Differences between the vehicle (top) and DV21 (bottom) groups are highlighted. (**b–d**) Summary histograms of seizure susceptibility, GTC episodes and rate of MC, respectively. With increasing DV21 concentrations, the incidence and severity of PTZ-induced seizures decreased. Two-way ANOVA was used. (**e**) Summary histograms of mortality. χ^2^ test was used. (n = 23, vehicle; n = 24, 300 mg/kg; n = 26, 600 mg/kg; n = 20, 900 mg/kg; n = 18, 1200 mg/kg). ***P *<* 0.001; **P < 0.01; *P < 0.05 versus to vehicle. Error bars are means ± s.e.m. NS, not detectable.

Pentylenetetrazole (PTZ), a GABA receptor antagonist, was used to induce the model of clonic seizures in healthy C57BL/6 mice^11^. PTZ (40 mg/kg; i.p.) induced seizures that progressed from hypoactivity (stage 1) to partial clonus (stage 2) and then to generalized clonus (stage 3), culminating in global tonic-clonic (GTC) seizures (stage 4). This type of model typifies the increasing severity stages of seizures, even leading to death in some cases^12^. We divided the mice into five groups. The first group received the vehicle, and the other four groups received various concentrations of DV21 corresponding to 300, 600, 900, and 1200 mg/kg. As the concentration increased, the severity of seizure behaviors significantly decreased after pretreatment with DV21 (Fig. 1a,b), the number of GTC seizures reduced (Fig. 1c), and the rate of myoclonic (MC) seizures reduced (Fig. 1d). Pretreatment with DV21 blocked seizure-related mortality, especially between the vehicle group and the 1200 mg/kg pretreatment group (Fig. 1e).

To measure electrographic changes induced by PTZ-induced seizures between the vehicle-treated mice and the DV21-treated mice, the electroencephalograms (EEGs) were recorded by using bilateral epidural screw electrodes (**see Methods**). Seven days after recovery from surgery, mice were administered PTZ, with or without 1 hr pretreatment with DV21. Both groups experienced myoclonic seizures and myoclonus stages, and the group that received the vehicle experienced longer and more severe GTC episodes (behaviors corresponding to stage 4), while the group pretreated with DV21 had fewer myoclonic seizures, with epileptic form and muscle activity being recorded by EEG and EMG (Fig. 1a). The results indicated that DV21 could decrease the frequency and severity of PTZ-induced mouse seizures and reduce seizure-related death. Furthermore, video-EEG monitoring also proved that the electrographic features of the PTZ-induced seizures were similar between the vehicle and DV21 treatment groups.

### DV21 decreases the seizure incidence and severity in a pilocarpine-induced mouse model

To induce status epilepticus (SE), the cholinergic agonist pilocarpine (250 mg/kg i.p.) which acts on muscarinic receptors^13^ was applied. The beginning of SE was defined as the onset of continuous generalized (stage 4 or 5) seizure activity^14^. To confirm the effect of DV21 on SE, we observed the period of stage 4 and 5 (the periods of GTC). The mice were also divided into five groups, similar to the PTZ groups. One group was injected with the vehicle as the control group; the other four groups were pretreated with different concentrations of DV21 before induction with pilocarpine.

**Figure 2:**
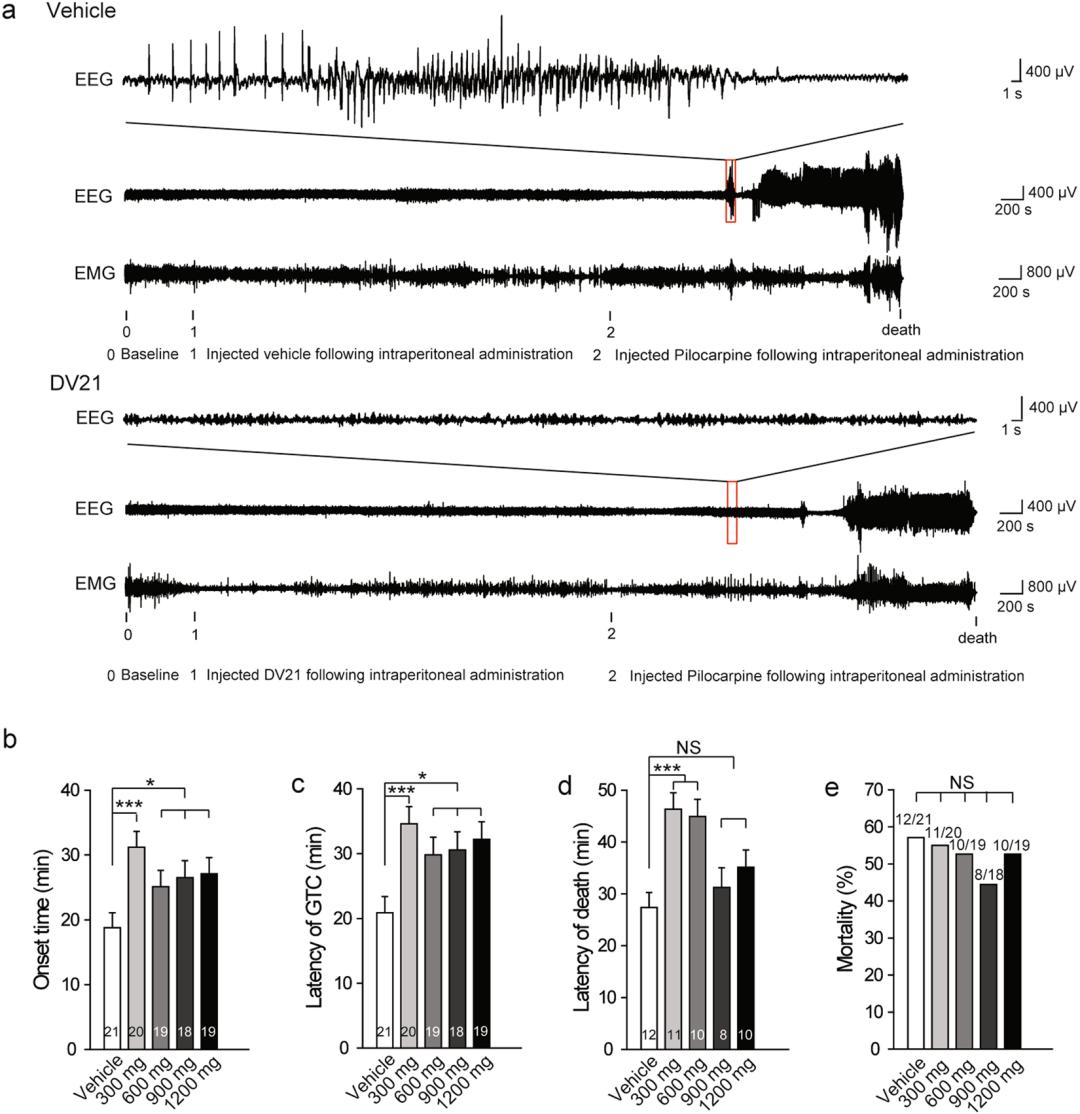
DV21 decreases pilocarpine-induced seizure incidence and severity. (**a**) Representative compressed electroencephalogram and electromyogram from a cortical lead depicting an electrographic seizure in a C57BL/6 mouse with seizure behaviors identified at the time of occurrence. Background EEG baseline is shown before seizure onset. Differences between the vehicle (top) and DV21 (bottom) groups are highlighted. (**b–d**) Summary histograms of the onset time, latency of GTC and latency of death, respectively. With increasing DV21 concentrations, the incidence and severity of pilo-induced seizures decreased. Two-way ANOVA was used. **(e)** Summary histograms of mortality. χ^2^ test was used. (n = 21, vehicle; n = 20, 300 mg/kg; n = 19, 600 mg/kg; n = 18, 900 mg/kg; n = 19, 1200 mg/kg). ***P *<* 0.001; *P < 0.05 versus to vehicle. Error bars are means ± s.e.m. NS, not detectable.

To observe the effect of DV21 on the spectrum of seizure activity, we monitored EEGs recordings, and the electrographic seizures were quantified by the latency onset time, the latency of GTC, the latency of death, and the mortality of seizures. We found that much more severe seizures were triggered in mice that had not been treated with DV21 (Fig. 2a). The results indicated that DV21 could reduce the onset time of epilepsy (Fig. 2b), especially at the dose of 300 mg/kg. Therefore, we analyzed the possible reason that might be related to the effective concentration of DV21 in the brains in this epilepsy model. The latency of GTC and death were significantly prolonged with DV21 treatments at concentrations of 300 mg/kg and 600 mg/kg (Fig. 2c,d). Moreover, we found mortality was slightly decreased, but there was no significant difference compared with the vehicle-treated control group (Fig. 2e).

### DV21 decreases the seizure incidence and severity in KA-induced mouse model

To further confirm the effect of DV21, we also tested another acute epilepsy model based on kainic acid (KA), which is an agonist of one class of glutamate receptors that is used to mimic the major neuropathological features of human temporal lobe epilepsy (TLE)^15^. In this model, mice were also injected with vehicle or different concentrations of DV21 before administration with KA (10 mg/kg, i.p.). Animals were injected with KA doses increasing could observe 5 stages: after the low doses, animals developed immobility to rigidity (stage 1–2); with further KA, animals developed automatisms with forelimb scratching, head bobbing, and circling behavior (stage 3); after the high doses, animals progressed to intermittent (stage 4) followed by continuous (stage 5) rearing with forelimb clonus and falling behavior^16^. In our studies, we could observe the stage 1–3 at the dose of 10 mg/kg of KA.

**Figure 3:**
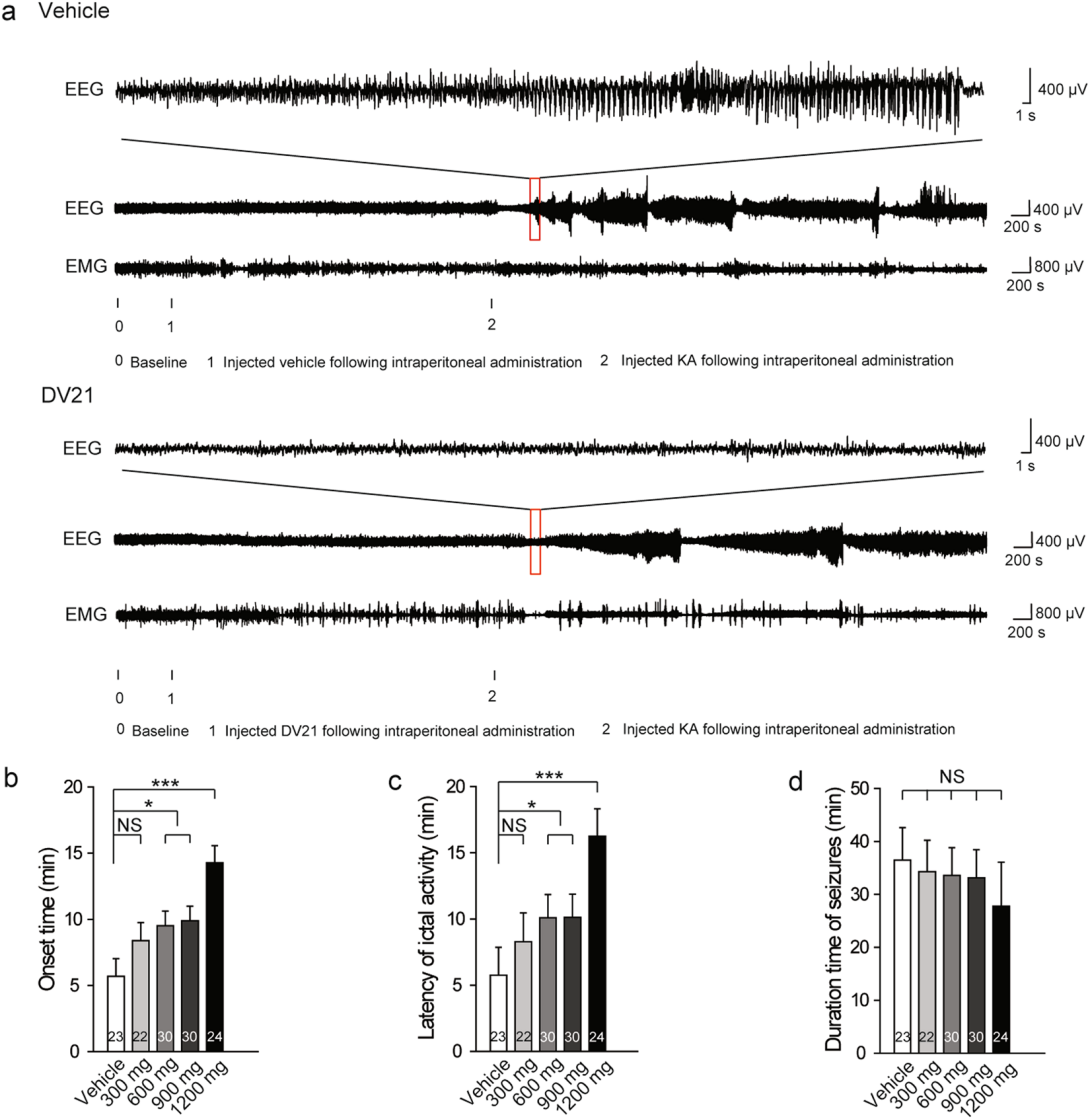
DV21 decreases KA-induced seizure incidence and severity. (**a**) Representative compressed electroencephalogram and electromyogram from a cortical lead depicting an electrographic seizure in a C57BL/6 mouse with seizure behaviors identified at the time of occurrence. Background EEG baseline is shown before seizure onset. Differences between the vehicle (top) and DV21 (bottom) groups are highlighted. (**b–d**) Summary histograms of the onset time, latency of ictal activity and duration time of seizures, respectively. With increasing DV21 concentrations, the incidence and severity of KA-induced seizures decreased. Two-way ANOVA was used. (n = 23, vehicle; n = 22, 300 mg/kg; n = 30, 600 mg/kg; n = 30, 900 mg/kg; n = 24, 1200 mg/kg). ***P *<* 0.001; *P < 0.05 versus to vehicle. Error bars are means ± s.e.m. NS, not detectable.

We observed that mice in both the vehicle and DV21-treated groups exhibited episodic seizures (Fig. 3a). EEGs quantification indicated that the onset-time and the latency of ictal activity were gradually increased after administration of different concentrations of DV21, especially at the dose of 1200 mg/kg (Fig. 3b,c). However, there were no significant differences in the duration of seizures among all the groups (Fig. 3d **)**.

Three different types of acute epilepsy models were used to imitate human seizures. We discovered that DV21 could significantly decrease seizure incidence and delay the onset of seizures and the seizure stages. However, further studies are required to investigate the role of DV21 in the chronic epilepsy models.

### Comparison of the effects of DV21 and two clinical drugs by intraventricular administration

**Figure 4:**
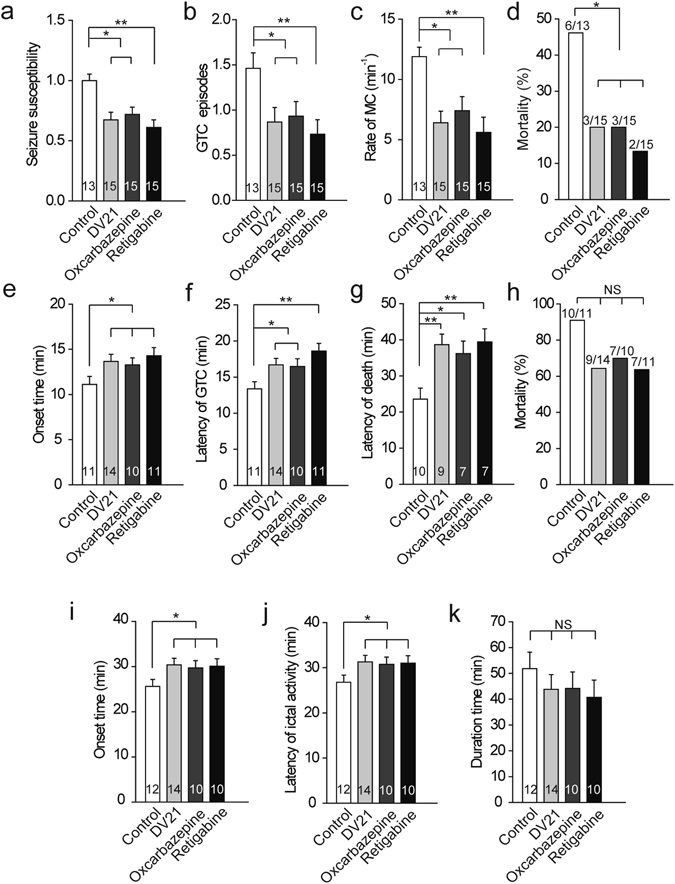
Comparison of the effect between DV21 and clinically administered drugs on drug-induced seizure models via intraventricular drug administration. (**a–c**) The effects of vehicle, DV21, oxcarbazepine or retigabine treatment on the PTZ-induced epilepsy models. Summary histograms of seizure susceptibility, GTC episodes and the rate of MC, respectively. Two-way ANOVA was used. (**d**) Summary histograms of mortality. χ^2^ test was used. (n = 13, control; n = 15, DV21; n = 15, oxcarbzepine; n = 15, retigabine). (**e–g**) Summary histograms of the onset time, latency of GTC and latency of death in the pilocarpine-induced seizure model after vehicle, DV21, oxcarbazepine or retigabine treatment. Two-way ANOVA was used. (**h**) Summary histograms of mortality. χ^2^ test was used. (n = 11, control; n = 14, DV21; n = 10, oxcarbzepine; n = 11, retigabine). (**i–k**) Comparison of the onset time, latency of ictal activity and seizure duration on the KA-induced seizure model after vehicle, DV21, oxcarbazepine or retigabine treatment. Two-way ANOVA was used. (n = 12, control; n = 14, DV21; n = 10, oxcarbzepine; n = 10, retigabine). **P < 0.01; *P < 0.05 versus to control. Error bars are means ± s.e.m. NS, not detectable.

To further confirm the treatment spectrum of DV21, we compared it with two clinically administered antiepileptic drugs (oxcarbazepine (50 ng) and retigabine (0.5 ng)) through intraventricular drug administration of three different types of acute epilepsy models (Fig. 4). It has been reported that retigabine is an antiepileptic drug (AED) for the treatment of partial-onset seizures and a positive allosteric modulator of M-type K^+^ channels^17^. Oxcarbazepine (OCBZ) is used to treat partial and generalized tonic-clonic seizures probably due to its effect on voltage-dependent sodium channels^18^.

Compared to the vehicle group, pretreatment with DV21 (4.7 ng), oxcarbazepine, or retigabine by intraventricular administration reduced seizure susceptibility, reduced the number of GTC seizures, reduced the rate of MC, and also blocked seizure-related mortality in the PTZ models (Fig. 4a–d). In pilocarpine-seizure models, DV21 also showed a similar effect to oxcarbazepine and retigabine, delaying the onset time and decreasing seizure severity; however, it showed no significant effects on seizure-related mortality **(**Fig. 4e–h **)**. Similar results were also observed in the KA-induced seizure models—the onset time and latency of ictal activity were reduced with pretreatment with DV21, oxcarbazepine and retigabine (Fig. 4i,j). However, there were no changes in the duration time of seizures **(**Fig. 4k **)**. Our studies indicated that, compared with different antiepileptic drugs (AEDs), which have their own mechanisms, DV21 showed a similar antiepileptic profile.

### Testing the concentrations of DV21 in the blood and brain

During the development of antiepileptic drugs, the *in vivo* anticonvulsant activity may be due to pharmacodynamics, but antiepileptic efficacy generally depends on pharmacokinetics^19^. We next measured the concentrations of DV21 in the serum and brain tissue. The results indicated that the concentration of DV21 in mouse plasma reached a peak value of 40.554 mg/ml at 1 h after intraperitoneal administration of DV21, and the distribution half-life (t_1/2α_) was 1.038 h (Supplementary Fig. 2a), whereas the concentration of DV21 in the mouse brain tissue reached a peak value of 37.07 ng/g at 6 h, and its distribution half-life (t_1/2α_) was 4.514 h (Supplementary Fig. 2b). The concentration-time curves were fitted to a two-compartment model. These pharmacokinetic parameters suggested that the distribution of DV21 *in vivo* was slow, and the elimination of DV21 was relatively slow after intraperitoneal administration.

### DV21 reduced the excitability of cortical pyramidal neurons partly by increasing the amplitude of M current

**Figure 5:**
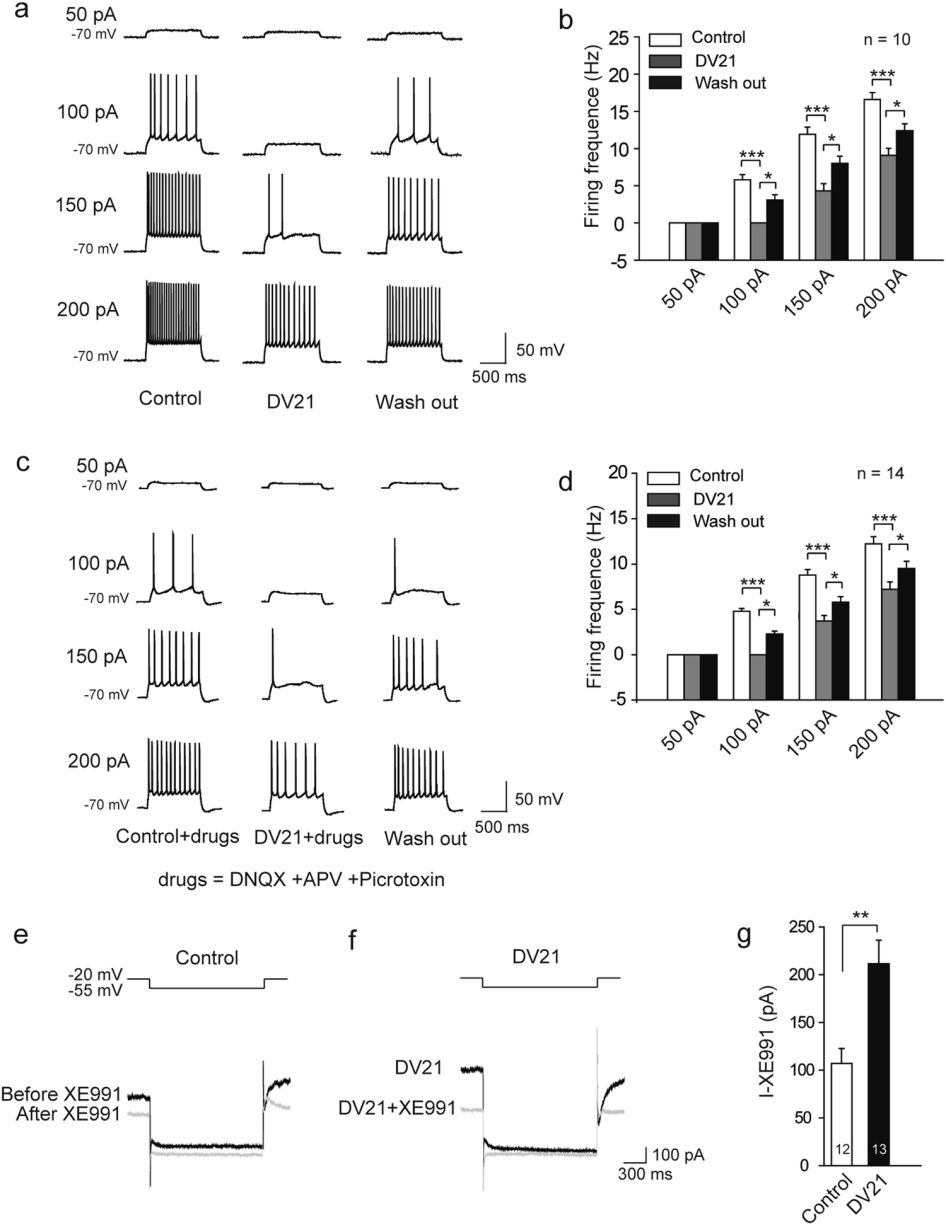
DV21 decreases the excitability of cortical pyramidal neurons partly by increasing M current. (**a**) Left, voltage responses of a representative to in pyramidal neurons to various injection steps (1s) from top to bottom, 50, 100, 150, 200 pA. Middle, same pyramidal neuron as in left panel after bath DV21for 15 minutes. Right, same pyramidal neurons as in middle after wash out. Current injections, from top to bottom, 50, 100, 150, 200 pA. Membrane potential was kept at −70 mV by injecting a small DC current through the recording pipette. (**b**) Summary histogram showing the effect of DV21 on the number of pyramidal neuron APs. Two-way ANOVA was used. n = 10. (**c**) Left, voltage responses of a representative to in pyramidal neurons to various injection steps (1 s) from top to bottom, 50, 100, 150, 200 pA. Middle, same pyramidal neuron as in left panel after the addition of DNQX, APV, and picrotoxin and DV21 for 15 minutes. Right, same pyramidal neurons as in middle after wash out. Current injections, from top to bottom, 50, 100, 150, 200 pA. Membrane potential was kept at −70 mV by injecting a small DC current through the recording pipette. (**d**) Summary histogram showing the effect of DV21 after the addition of DNQX, APV, and picrotoxin on the number of pyramidal neuron APs. Two-way ANOVA was used. n = 14. (**e**,**f**) The M current protocol and representative M current traces. M current were induced by a step hyperpolarization (1.5 s) to −55 mV from a holding potential of −20 mV in pyramidal neurons from control (**e**) and DV21 (**f**) groups before and after the application of 20 µM XE991. (**g**) Summary histogram showing the effect of DV21 on Mcurrent. (n = 12, Control; n = 13, DV21.) *P*-value was calculated by two-sided *t*-test. ***P *<* 0.001; **P < 0.01; *P < 0.05. Error bars are means ± s.e.m.

Seizures are abnormal discharges in the cerebral cortex^20^. To investigate the mechanism underlying the antiepileptic effects of DV21, we recorded the excitability of layer 5/6 cortical pyramidal neurons in acute cortical slices by examining the number of action potentials (APs) elicited by current injections of various amplitudes (1 s, 0 to +250 pA). As the amplitude of the injected current increased, all pyramidal neurons showed high-frequency discharges. However, the number of APs was significantly reduced after the administration of DV21 compared with the control and wash-out groups. The inhibition effect was evident within 15 min of DV21 application and disappeared approximately 10 min after the removal of DV21 (Fig. 5a,b). To obtain the EC_50_ of DV21 on cortical pyramidal neurons, different concentrations of DV21 were applied, and we found the concentration of 10 μM as the EC_50_ (Supplementary Fig. 3). The effect of DV21 on pyramidal neuron excitability could be caused by two possibilities: one is the excitatory or inhibitory input to pyramidal neurons, and the other is the direct modulation of the intrinsic excitability of pyramidal neurons^21^. To distinguish between the two possibilities, DL-2-amino-5-phosphonovaleric acid (DL-AP5; 50 μM), 6, 7-dinitroquinoxaline-2, 3-dione (DNQX; 20 μM) and picrotoxin (100 μM) were applied to block NMDA-mediated, AMPA-mediated and GABA_A_-mediated synaptic transmission, respectively. The results showed that the effects of DV21 remained (Fig. 5c,d), suggesting that DV21 directly affected the intrinsic properties of pyramidal neurons.

To further investigate the intrinsic properties of cortical pyramidal neurons, we analyzed the action potential parameters before and after adding DV21 to the extracellular solution. We performed whole-cell current-clamp recordings and measured their membrane properties (Supplementary Fig. 4). Our results indicated that DV21 increased the half-width (Supplementary Fig. 4c) and AHP amplitude (Supplementary Fig. 4d). However, the results showed that there were no significant changes in other spike parameters, including threshold (Supplementary Fig. 4a) and amplitude (Supplementary Fig. 4b) after DV21 treatment. Upon further identification of cortical pyramidal neurons by measuring their membrane properties, we found that the resting membrane potential (RMP) was reduced after DV21 treatment (Supplementary Fig. 4e), but there were no significant differences in other membrane properties, including membrane capacitance (Cm) (Supplementary Fig. 4f), input resistance (Rm) (Supplementary Fig. 4g), and membrane time constant (τ_m_) (Supplementary Fig. 4h). The results indicated that DV21 treatment decreased the RMP and suggested that it might affect the passive membrane properties of cortical pyramidal neurons.

Considering that potassium channels play fundamental roles in the regulation of membrane excitability^22^, the M current are thought to play a pivotal role in mediating excitability control. We hypothesized that M-channels may be a target for the modulation of cortical pyramidal neuron excitability by DV21. To confirm this hypothesis, we measured M current by first voltage-clamping neurons to −20 mV and inactivating delayed rectifier potassium channels, followed by hyperpolarization to −55 mV for deactivation^23^. Then, we repeated this protocol in the presence of 20 μM XE991. This concentration almost completely blocked the M-channels^24^. We defined the M current as the XE991-sensitive difference current (Fig. 5e,f). Bath application of DV21 significantly increased M current in the cortical pyramidal neurons (Fig. 5f,g). Furthermore, we recorded M current at different concentrations of DV21. The results indicated that DV21 could increase the amplitude of M current by increasing concentrations (Supplementary Fig. 5).

Further, to see the effect of DV21 when only partially inhibit the M current, we used a concentration of 10 μM linopridine that inhibits 75–90% of M current in neurons^9^. The results also indicated that DV21 could also increase the amplitude of M current (Supplementary Fig. 6).

Next, we investigated the contributions of DV21 to medium afterhyperpolarizations (mAHPs), which is a conductance that requires the presence of M-channels^9^. We elicited mAHP following a series of spikes by injecting a 50-ms depolarizing current pulse into the pyramidal neurons (Supplementary Fig. 7). To exclude the interference from calcium-activated SK channels, we added the SK channel specific blocker apamin (100 nM) to the extracellular solution. Compared with the control group, the amplitude of mAHPs increased almost two-fold after treatment with DV21 (Supplementary Fig. 7a–d). Application of 20 µM XE991 reduced the amplitude of mAHPs by ~85%, and minimal changes were observed after the treatment with DV21 (Supplementary Fig. 7e–h).

These results indicated that DV21 influences the excitability of cortical pyramidal neurons partly by increasing M current.

### XE991 blocked most the effect of DV21 *in vivo* and in *vitro*

**Figure 6:**
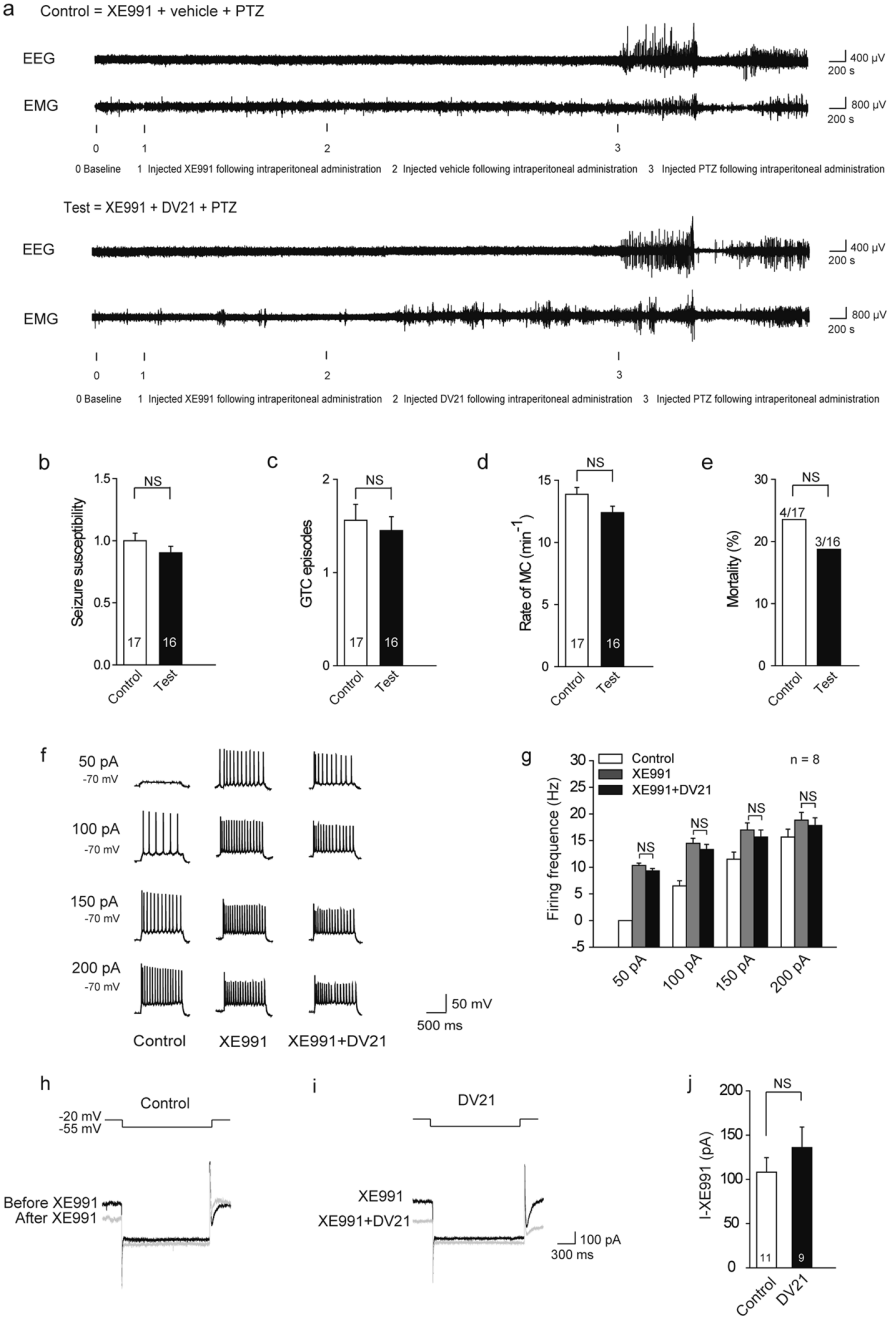
XE991 blocked most effect of DV21 in the PTZ-induced seizure model and on pyramidal neurons. (**a**) Representative compressed electroencephalogram and electromyogram from a cortical lead depicting an electrographic seizure in a C57BL/6 mouse with seizure behaviors identified at the time of occurrence. Background EEG baseline is shown before seizure onset. (**b–e**) Summary histograms of seizure susceptibility, GTC episodes, rate of MC and mortality, respectively. (n = 17, control; n = 16, test.) *P*-value was calculated by two-sided *t*-test. (**f**) Left, voltage responses of a representative to in pyramidal neurons to various injection steps (1 s) from top to bottom, 50, 100, 150, 200 pA. Middle, same pyramidal neuron as in left panel after bath of 20 µM XE991 for 30 minutes. Right, same pyramidal neurons as in middle after wash out of XE991 + DV21. Current injections from top to bottom, 50, 100, 150, 200 pA. Membrane potential was kept at −70 mV by injecting a small DC current through the recording pipette. (**g**) Summary histogram showing the effect of DV21 after the addition of XE991 on the number of pyramidal neuron APs. Two-way ANOVA was used. n = 8. (**h**) The M current protocol and representative M current traces. The M current was induced by a step hyperpolarization (1.5 s) to −55 mV from a holding potential of −20 mV in pyramidal neurons from before and after application of 20 μM XE991 in the control group. (**i**) The M current was induced in the group to which XE991 was added before DV21. (**j**) Summary graph showing the effect of DV21 on M current after blocking M-channels. (n = 11, Control; n = 9, DV21.) *P*-value was calculated by two-sided *t*-test. NS, not detectable.

To further confirm that DV21 regulates the activity of cortical pyramidal neurons through M current, we first blocked M-channels with XE991 (2.5 mg/kg, i.p.)^25^ 45 minutes before application of DV21 in the PTZ-induced epilepsy models (Fig. 6a). The results indicated that there were no significant differences in seizure susceptibility (Fig. 6b), GTC episodes (Fig. 6c), the rate of MC (Fig. 6d) or mortality (Fig. 6e) between the control and test groups.

Next, to further demonstrate the role of M current in the effect of DV21, we first blocked M-channels before administration of DV21 to the pyramidal neurons. Upon treatment with bath application of 20 μM XE991 for 30 minutes, the pyramidal neuron excitability significant increased, and the excitability exhibited minimal changes after 20 minutes of treatment with DV21 (Fig. 6f,g). Moreover, we also recorded the M current, and there were no significant changes between the control and DV21 groups (Fig. 6h–j). These results further indicated that M current may play a critical role in the regulation of excitability of pyramidal neurons by DV21.

## Discussion

In this study, we provided evidences that a natural product DV21 could decrease the incidence and severity of seizures through M current. The main findings of this study are as follows. First, we found that the anticonvulsant natural triterpenoid DV21 could improve the symptoms of epilepsy, which was identified from traditional Chinese medicine (TCM). Second, DV21 prolonged the latency and decreased the incidence and severity of seizures in PTZ-, pilocarpine- and KA-induced acute epilepsy models, but the effect was most inhibited by applying the M-channel inhibitor XE991 before treatment with DV21. Third, DV21 could reduce the excitability of cortical pyramidal neurons, and this effect disappeared after blockade of M-channels, suggesting that DV21 might regulate the activity of pyramidal neurons through M current. In summary, our study identified that DV21 could reduce the symptoms of epilepsy and that M-channels may be a target of DV21 in the regulation of cortical pyramidal neuron excitability. These findings suggest that DV21 may become a new prospect for the treatment of epilepsy.

It has been shown that traditional pharmacological approaches to treat epilepsy have side effects, tolerability issues and pharmacokinetic disadvantages that render their use problematic^26^. We identified DV21, which is a natural product and has been proved pharmacologically safe in previous publications, by high-throughput screening in a zebrafish seizure model. We tested the effect of DV21 on mobility and found that with the dose of 1200 mg/kg (*p* > 0.05, n = 20) and 2000 mg/kg (*p* > 0.05, n = 15), there were no significant differences between the control and the DV21 groups within 30 minutes. These results suggested that treatment of DV21 at the dose of 1200 mg/kg and 2000 mg/kg by intraperitoneal administration should not cause immobility (Supplementary Fig. 8). The effect of DV21 was concentration-dependent, and even at high concentrations, it did not induce obvious side effects. Pharmacokinetic evaluation revealed the efficacy of the drug in preclinical trials^19^, and our study indicated that DV21 has ideal pharmacokinetic properties in blood and brain tissue. Future studies should investigate whether DV21 alone has any effects on the animals’ innate behavior besides mobility and test for the adverse side effects. In addition, the metabolism of DV21should also be further studied.

Different animal models of epilepsy represent different causal mechanisms underlying the disease in humans^11^. In the present study, we applied three acute epilepsy models to test the anticonvulsant effects of DV21. All the results suggested that DV21 could reduce epilepsy symptoms upon intraperitoneal injection and intraventricular administration in different models. The effect was blocked after applying the M-channels inhibitor XE991 before intraperitoneal injection of DV21 in PTZ-induced seizure models. Compared with two clinical drugs, the treatment spectrum of DV21 is similar to retigabine. These results suggested that DV21 exerted effects for the treatment of epilepsy in acute epilepsy models, and further studies should confirm its effects in chronic epilepsy models.

Cortical pyramidal neurons are the most excitatory cells in the mammalian cortex^27^. Seizures can be generated in response to a loss of balance between excitatory and inhibitory influence^28^. Our results suggested that DV21 decreased seizure susceptibility, unlike other factors involved, such as decreased GABA_A_ expression, changes in HCN-channel expression, or activation of inflammatory responses^29^. Electrophysiology studies suggested that DV21 could decrease the excitability of cortical pyramidal neurons, and we provided evidence that DV21 directly changed the intrinsic properties of cortical pyramidal neuron and that this effect was not regulated by synaptic inputs because the effects of DV21 remained when picrotoxin, DL-AP5 and DNQX were applied. Changes in the intrinsic properties of pyramidal neurons included increased amplitudes of AHP and decreased resting membrane potentials of the action potential. M-channels are widely expressed in peripheral and central neurons^10^ and underlie M current. It has been shown that M current play a pivotal role in regulating the membrane excitability of various central and peripheral neurons^30, 31^. In both humans^32^ and animal models^10^, the suppression of neuronal M current could result in epileptic conditions. Our results indicated that DV21 decreased the excitability of cortical pyramidal neurons by increasing M current. This conclusion is further supported by the increased excitability of cortical pyramidal neurons upon blocking M-channels prior to treatment with DV21. M-channels are thought to generate mAHPs during and after repetitive neuronal discharge^33^, and therefore, we also investigated the contribution of DV21 to mAHPs in current-clamp recordings from cortical pyramidal neurons. M-channels mediated mAHPs, and pyramidal cells showed much bigger mAHPs after treatment with DV21. Blocking M-channels led to a substantial loss of mAHPs, and there was no significant change after the addition of DV21. The results also showed that DV21 could increase the spike width, so it suggested that DV21 might have effects on other ion channels other than the M-channels. Future work is required to investigate whether DV21 exerts effects on other neurons and other ion channels.

Overall, both the *in vivo* behavioral tests and *in vitro* electrophysiological recordings reported in the current study revealed a new natural product that could decrease the severity of epilepsy and identified M current may as one molecular target of DV21 in the regulation of cortical pyramidal neuron excitability. However, further preclinical evaluations should be conducted to evaluate the adverse effects of long-term administration of DV21. Our findings suggest that DV21 might be a new anticonvulsant drug to treat epilepsy.

## Methods

All animal-related experimental procedures were reviewed and approved by the Animal Advisory Committee at Zhejiang University in accordance with the National Institutes of Health Guidelines for the Care and Use of Laboratory Animals.

### Isolation and identification of DV21

#### General Experimental Procedures

Optical rotations were measured on a P-1020 polarimeter (JASCO, Tokyo, Japan). IR spectra were detected on a Bruker Tensor 27 spectrometer with KBr pellets. UV data were obtained on a Shimadzu UV2401PC spectrophotometer. 1D- and 2D-NMR spectra were recorded on Bruker DRX-500 spectrometers operating at 500 MHz for ^1^H NMR spectrum, and 125 MHz for ^13^C NMR spectrum. Coupling constants are expressed in Hertz and chemical shifts are given on a ppm scale with reference to the solvent signals. ESIMS were performed on Waters Xevo TQ-S. HREIMS were recorded on an API Qstar time-of-flight spectrometer and on a Waters Auto Spec Premier P776 mass spectrometer. HRESIMS were recorded on an API QstarPulsa LC/TOF spectrometer. HPLC analysis was run on an Angilent 1260 liquid chromatography. Column chromatography (CC) was performed with silica gel (200–300 mesh, Qingdao Haiyang Chemical Co., Ltd., Qingdao, People’s Republic of China), LiChroprep RP-18 gel (40–63 *μ*m, Merck, Darmstadt, Germany) and MCI-gel CHP-20P (Mitsubishi Chemical Co., Tokyo, Japan). Thin-layer chromatography (TLC) was carried out on silica gel H-precoated plates (Qingdao Haiyang Chemical Co., Ltd., Qingdao, People’s Republic of China). Spots were detected by spraying with 10% H_2_SO_4_ in EtOH followed by heating. Preparative HPLC was performed on a Gilson liquid chromatography with a 7 *μ*m Zorbax SB-C_18_ (21.2 × 250 mm) column. Semi-preparative HPLC separation was performed on an Agilent 1260 liquid chromatography with a 5 *μ*m Thermo BDS HYPERSIL-C_18_ column (10 × 250 mm).

#### Plant Material

The stem of *Dregea volubilis* (L. f.) Benth. ex Hook. f. was collected from Yunnan Province, People’s Republic of China, on June 2008, and were identified by Prof. Chong-Ren Yang from the State Key Laboratory of Phytochemistry and Plant Resources in West China, Kunming Institute of Botany, Chinese Academy of Sciences. A voucher specimen was deposited at the State Key Laboratory of Phytochemistry and Plant Resources in West China, Kunming Institute of Botany, Chinese Academy of Sciences.

#### Extraction and Isolation

The air-dried stems of *D. volubilis* (5.5 kg) were powdered and extracted with MeOH at 60 °C. After removal of the solvent under reduced pressure, the MeOH extract was partitioned between CHCl_3_ and H_2_O (1:1, v/v) to furnish an CHCl_3_–soluble fraction (168 g). The CHCl_3_ extract was applied to a silica gel column chromatography (CC), eluting with CHCl_3_–MeOH–H_2_O (86:17:1, v/v), to give fractions A–C. Fr. C (21 g) were chromatographed over MCI-gel CHP-20P and Rp-18 column, followed by preparative and semi-preparative HPLC (CH_3_CN–H_2_O, 35:65, v/v) to yield compound DV21 (40 mg). In order to get an amount of DV21 for animal bioassay, we isolated more than 10 g of DV21from leaves of *Eriobotrya japonica* according to the modified above procedure.

*The identification of DV21*: C_30_H_48_O_4_, white powder, ESI-MS *m/z*: 471[M-H]^−^, ^1^H-NMR (pyridine-*d*_5_, 500 MHz): δ_H_ 1.06 (3 H, s, Me-23), 0.93 (3 H, s, Me-24), 1.24 (3 H, s, Me-25), 1.00 (3 H, s, Me-26), 1.26 (3 H, s, Me-27), 0.97 (3 H, d, Me-29), 0.98 (3 H, d, Me-30), 5.45 (1 H, s, H-12), 3.37 (1 H, d, J = 9.3 Hz, H-3), 3.92 (1 H, m, H-2), 3.28 (1 H, m, H-18); ^13^C-NMR (pyridine-*d*_5_, 125 MHz): δc 48.2 (C-1), 69.0 (C-2), 84.2 (C-3), 40.3 (C-4), 56.3 (C-5), 19.3 (C-6), 33.6 (C-7), 40.2 (C-8), 48.6 (C-9), 38.9 (C-10), 24.1 (C-11), 122.9 (C-12), 145.3 (C-13), 42.4 (C-14), 28.7 (C-15), 24.4 (C-16), 47.1 (C-17), 42.6 (C-18), 46.8 (C-19), 31.4 (C-20), 34.6 (C-21), 33.6 (C-22), 29.8 (C-23), 18.2 (C-24), 17.3 (C-25), 17.9 (C-26), 26.6 (C-27), 180.7 (C-28), 33.7 (C-29), 24.2 (C-30).

*The purity analysis of DV21*: Liquid chromatography under isocratic conditions was performed a Waters Alliance (Waters, USA) quaternary pump equipped with an autosampler and diode array detection (DAD) system. A Millennium®^32^ system was used for data analysis. An Agilent ZORBAX SB-C_18_ column (4.6 × 15 0 mm, 5 *μ*m) was used. The following gradient system was used with acetonitrile (solvent A) and water containing 0.34% (v/v) formic acid (solvent B) with isocratic elution of the percentage of solvent A in solvent B 78%. The flowing rate was 1.0 mL/min; sample injection was 10 μL. Diode array detection was between 250 and 650 nm and the column temperature was set at 30 °C and the monitored wavelength was 203 nm. The retention time (Rt) of DV21 was 24.635 min. The purity of DV21 was more than 98.5%.

### Bioassay of seizure-like locomotor activity caused by pentylenetetrazole in zebrafish *Zebrafish*

A breeding stock of healthy mature zebrafish (4 to 5 pairs) was used for naturally embryos production. Each pair can yield up to 200 to 300 embryos and the dead embryos were removed 6 and 24 hours post-fertilization (hpf). The rest suitable embryos, according to the embryonic stage of development, were selected and then incubated in the fish water (1 L reverse osmosis water containing soluble salt (200 mg), with conductivity of 480–510 *μ*S/cm, pH 6.9–7.2, and hardness of 53.7–71.6 mg/L CaCO_3_) under 28 °C. Since the embryos can obtain nutrients from their own yolk sac, it is not necessary to feed them for nine days post-fertilization (dpf). The 3 dpf larval zebrafish were used for the antithrombotic test. Hunter Biotechnology, Inc. is accredited by the Association for Assessment and Accreditation of Laboratory Animal Care (AAA LAC) International. *Bioassay of seizure-like locomotor activity:* The pentylenetrazole (PTZ) was used to induce seizure-like locomotors of larval zebrafish as model group. The DV21 was added as the test group with a final concentration of 30 μM. The negative control group was the solvent group, which not only represented the background value of the test group, but also made sure that the actions of larval zebrafish were normal. Blank control group was used to prove that the solvent did not have harmful effects on the larval zebrafish. Phenytoniumnatricum (PHT), carbamazepine (CBZ), sodium valprotate (VPA), levetiracetam (LEV) and Retigabine (RTG) were used as the positive control. The experiment was carried on 96-well cell culture plates, on which there was a blank control, a solvent control, a model, a positive control and the test samples’ groups. Each group processed 8 wells with 4 larval zebrafish in each well. The quantitative analysis of the speed and distance of the faster moving (>20 mm/sec) was carried on a Danio Vision (V3, View Point Life Sciences Company) and analyzed by Nikon NIS-Elements D3.10 software. The inhibition ratio was calculated by the following equation, and before calculations the *D* (*test group*) and *D* (*model group*) should minus the background values. The results were shown as $\overline{X}$ ± SE. Statistical evaluation included one-way analysis of variance followed by Dunnett’s *t*-test for multiple comparisons. *P* < 0.05 (*) was taken as significant.

$${\rm{I}}{\rm{n}}{\rm{h}}{\rm{i}}{\rm{b}}{\rm{i}}{\rm{t}}{\rm{i}}{\rm{o}}{\rm{n}}{\rm{r}}{\rm{a}}{\rm{t}}{\rm{i}}{\rm{o}}({\rm{ \% }})=\frac{{\rm{D}}({\rm{m}}{\rm{o}}{\rm{d}}{\rm{e}}{\rm{l}}{\rm{g}}{\rm{r}}{\rm{o}}{\rm{u}}{\rm{p}})-{\rm{D}}({\rm{D}}{\rm{V}}21{\rm{g}}{\rm{r}}{\rm{o}}{\rm{u}}{\rm{p}})}{{\rm{S}}({\rm{m}}{\rm{o}}{\rm{d}}{\rm{e}}{\rm{l}}{\rm{g}}{\rm{r}}{\rm{o}}{\rm{u}}{\rm{p}})-{\rm{S}}({\rm{s}}{\rm{o}}{\rm{l}}{\rm{v}}{\rm{e}}{\rm{n}}{\rm{t}}{\rm{g}}{\rm{r}}{\rm{o}}{\rm{u}}{\rm{p}})}\times 100{\rm{ \% }}$$

### Mice

Experiments were completed with C57BL/6 mice. For all acute drug-induced epilepsy models, we used ten-week-old male C57BL/6 mice weighing between 25–30 g. By using behavior test, the mice were maintained in a 12/12 h light/dark cycle, under controlled environmental conditions. The mice that are with abnormal weight and appearance in the behavior test were excluded. No sexually dimorphic observations were made.

### Preparation of ***in vitro*** brain slices

The mice were prepared from P15 to P20 and they were deeply anesthetized by using isoflurane and rapidly killed by decapitation. The brain was quickly removed and the coronal slices (300 *μ*m) were prepared with a Vibroslice (Leica VT 1000S) in ice-cold artificial cerebrospinal fluid (ACSF) consisting of 125 mM NaCl, 3 mM KCl, 1.25 mM NaH_2_PO_4_, 2 mM MgSO_4_, 2 mM CaCl_2_,25 mM NaHCO_3_ and 10 mM glucose. Slices were stored at 34 °C for ~30 min, followed by ~60 min at room temperature. The solution was bubbled with 95% O_2_/5% CO_2_ to maintain a pH around 7.4.

### Electrophysiology

Neurons were visually identified under an upright microscope equipped with a 40X water- immersion lens (Nikon, ECLIPSE FN1) and recorded by using whole-cell techniques (MultiClamp 700B Amplifier, Digidata 1440A analog-to-digital converter) and pClamp 10.2 software (Axon Instruments/Molecular Devices). We recorded pyramidal neurons from layers V/VI in the PFC. Glass pipettes (3–4.5 MΩ) were filled with internal recording solution for whole-cell recording: 110 mM K-gluconate, 40 mM KCl, 10 mM HEPES, 2 mM Mg_2_ATP, 0.5 mM NaGTP, and 0.2 mM EGTA; pH was adjusted to 7.25 with 10 M KOH. Recording the M current, the extracellular solution also included TTX (500 nM; Sigma), 4-AP (2 mM), CsCl (1 mM), CdCl_2_ (100 µM), and apmin (100 nM) to block voltage-gated sodium channels, calcium channels, calcium-activated SK channels, HCN channels, and A-type potassium channels, respectively. We also added DNQX (20 µM, Tocris Bioscience), APV (50 µM, Tocris Bioscience), and picrotoxin (100 µM) (Abcam) to block AMPA-mediated, NMDA-mediated, and GABA-mediated synaptic transmission. To analyze the passive and active membrane properties of the neurons before and after adding the DV21, we recorded twice the amplitude of RMP, input resistance (*R*_in_), membrane time constant (τ_m_), and membrane capacitance (*C*m), respectively. We defined that the RMP are the average membrane potential within 2 min after obtaining the whole-cell configuration and 15 min after the DV21 administration in the extracellular solution. Input resistance (*R*_in_) was calculated by rectangular hyperpolarizing current injections (1–50 pA) before and after adding the DV21. Membrane time constant (τ_m_) was recorded by fitting a single exponential function to these same hyperpolarizing voltage deflections. Membrane capacitance (*C*m) was obtained by dividing τ_m_ by *R*_in._ We assessed the spike properties with the first spike evoked by a small suprathreshold current step applied from RMP before and after adding DV21. Voltage threshold was defined as d*V*/d*t* *=* 10 mV ms^−1^. Spike amplitude was measured as the voltage difference between the AP peak and the AP threshold. Afterhyperpolarization was assessed by the difference between the spike threshold and minimum voltage after the AP peak. Spike width was obtained from half the spike amplitude.

### Analysis of DV21 and its metabolites concentration in blood and brain

#### Dissolve the DV21

We added the 20% Tween 80 to hydrotropy DV21 dissolved in normal saline, getting the final concentration of 600 mg/kg.

#### Instrument and analytical conditions

The analysis was carried out on an Agilent 1260 series LC system with a binary pump, autosampler and an Agilent 1260 Series LC/MSD SL (6410B) quadrupole mass spectrometer. The separation was performed at 30 °C using an Agilent ZORBAX SB-C_18_ column (2.1 × 100 mm, 3.5 *μ*m). The solvents were acetonitrile (solvent A) and 0.1% (v/v) aqueous formic acid (solvent B). Isocratic elution was 25% A in 5 min at a flow rate of 0.2 mL/min. Under these conditions, the retention times for DV21 was 1.35 min.

The detection was carried out with electron spray ionization (ESI) operation at positive ion and multiple reactions monitoring (MRM) model. The other parameters were as follows: Gas temp: 350 °C; Gas flow: 150 L/hr; nebulizer: 7.0 bar; capillary voltage: 4000 v. Nitrogen was used as the nebulizer gas, dry gas and collision gas. Mass spectra were obtained as a dwell time of 0.3 s.

#### Sample preparation procedure

**Blood**: We divided mice into different groups and injected DV21 (600 mg/kg), we removed the eyeball blood when the time was up. Next, we collected the supernatants after a 10 minutes 4000 rpm centrifugation. **Brain**: Divided mice into different groups with equal mice in each group and injected DV21 (600 mg/kg), then we removed the brain quickly after perfusion with 0.9% saline. Next, we homogenized the tissue in the PBS buffer and collected the supernatants after a 10 minutes 15,000 rpm centrifugation.

### Surgeries and EEG measurements

After we anesthetized ten-week-old mice by isoflurane, they were secured in a stereotactic head frame and made an incision along the midline. We used 8% H_2_O_2_ to display the bregma and posterior, then we leveling around. The electrode was placed over the cranium with three screws (at 3 positions: the first screw (left): A-P: +1.5 mm, lateral: +1.5 mm; the second and the third screws: A-P: −3 mm, lateral: ±3 mm). The cannula (intraventricular drug administration) was placed into the intraventricular (position (right): A-P: −0.22 mm, lateral: −1.0 mm depth: −2.5 mm). One week after the surgery, we recorded EEGs and EMGs in freely moving mice. We defined the amplitude that is more than 400 mV as abnormal wave.

### Drug-induced seizures and monitoring

We assessed the PTZ-induced seizures through 4 stages: Stage *1*: Hypoactivity culminating in behavioral arrest with contact between abdomen and the cage. Stage *2*: Partial clonus (PC) involving the face, head, or forelimbs. Stage *3*: Generalized clonus (GC) including all four limbs and tail, rearing, or falling. Stage *4*: Generalized Tonic-Clonic seizure (GTC)^12^. We calculated the seizure susceptibility from the latencies to stage 2–4 and the formula was:

$$\begin{array}{c}{\rm{S}}{\rm{u}}{\rm{s}}{\rm{c}}{\rm{e}}{\rm{p}}{\rm{t}}{\rm{i}}{\rm{b}}{\rm{i}}{\rm{l}}{\rm{i}}{\rm{t}}{\rm{y}}\,{\rm{s}}{\rm{c}}{\rm{o}}{\rm{r}}{\rm{e}}\,=\,\,{\sum (0.2\ast \frac{1}{{\rm{l}}{\rm{a}}{\rm{t}}{\rm{e}}{\rm{n}}{\rm{c}}{\rm{y}}{\rm{t}}{\rm{o}}{\rm{P}}{\rm{C}}}+0.3\ast \frac{1}{{\rm{l}}{\rm{a}}{\rm{t}}{\rm{e}}{\rm{n}}{\rm{c}}{\rm{y}}{\rm{t}}{\rm{o}}{\rm{G}}{\rm{C}}}+0.5\ast \frac{1}{{\rm{l}}{\rm{a}}{\rm{t}}{\rm{e}}{\rm{n}}{\rm{c}}{\rm{y}}{\rm{t}}{\rm{o}}{\rm{G}}{\rm{T}}{\rm{C}}})}^{}\end{array}$$

We assessed the KA- and pilo- induced seizures according to following methods: Onset time is measured from the moment when the C57BL/6 mice were injected with KA or pilocarpine, until the first epileptic waves. Latency of GTC is measured from the moment of pilocarpine injection in the C57BL/6, until the first generalized tonic-clonic seizure epileptic waves. Latency of death is measured from the moment injection pilocarpine, until the death of the mice. Latency of ictal activity is measured from the moment of KA injection, until the first continuous frequency and amplitude increased waves. Duration time of seizures is the sum total of all seizures time.

### Statistics

Unless otherwise specified, data are expressed as mean ± s.e.m.; error bars also indicate s.e.m. (n = number of individual samples). For comparison of means from the same group of cells, a two-tailed Student’s *t*-test was used. Differences between more than two group designs were tested with two-way analysis of variance (ANVOA) respectively. For comparing different mortality between samples, Pearson’s χ^2^ test was applied. Differences were considered significant if *P* < 0.05.

## Electronic supplementary material

Supplementary Information
