# Supplementary material for: DV21 decreases excitability of cortical pyramidal neurons and acts in epilepsy
Source: Sci Rep. 2017 May 10;7:1701. doi: 10.1038/s41598-017-01734-z (PMC5431874; doi:10.1038/s41598-017-01734-z)
Supplement: Supplementary file 1 — Supplementary Information [file 41598_2017_1734_MOESM1_ESM.pdf]

Supplementary Information

DV21 decreases excitability of cortical pyramidal neurons and acts in epilepsy

Min Xu[1,2,3,+,*], Peng Sun[1,+], Ying Zhang[1],Ci-Hang Yang[1], XinWei[2], Xiao-Xia Ma[2], Kun-Ming Ni[1], Chong-Ren Yang[2], Ying-Jun Zhang[2], Xiao-Ming Li[1*]

[1]Department of Neurobiology, Institute of Neuroscience, Key Laboratory of Medical Neurobiology of the Ministry of Health of China, Joint Institute for Genetics and Genome Medicine between Zhejiang University and University of Toronto, Collaborative Innovation Center for Brain Science, Zhejiang University School of Medicine, Hangzhou, Zhejiang, China.

[2]State Key Laboratory of Phytochemistry and Plant Resources in West China, Kunming Institute of Botany, Chinese Academy of Science, Kunming, China.

[3]Center for Pharmaceutical Sciences, Faculty of Life Science and Technology, Kunming University of Technology and Science, Kunming, China.

[+]These authors contributed equally to this work.

*****Correspondence should be addressed to M. X. (xumin8121@hotmail.com) or X.-M. Li. (lixm@zju.edu.cn), Department of Neurobiology, Key Laboratory of Medical Neurobiology of Ministry of Health of China, Zhejiang University School of Medicine, 866 Yu-Hang-Tang Road, Hangzhou, 310058, China.

Tel: 86-571-8820-8757; Fax: 86-571-8820-8757.

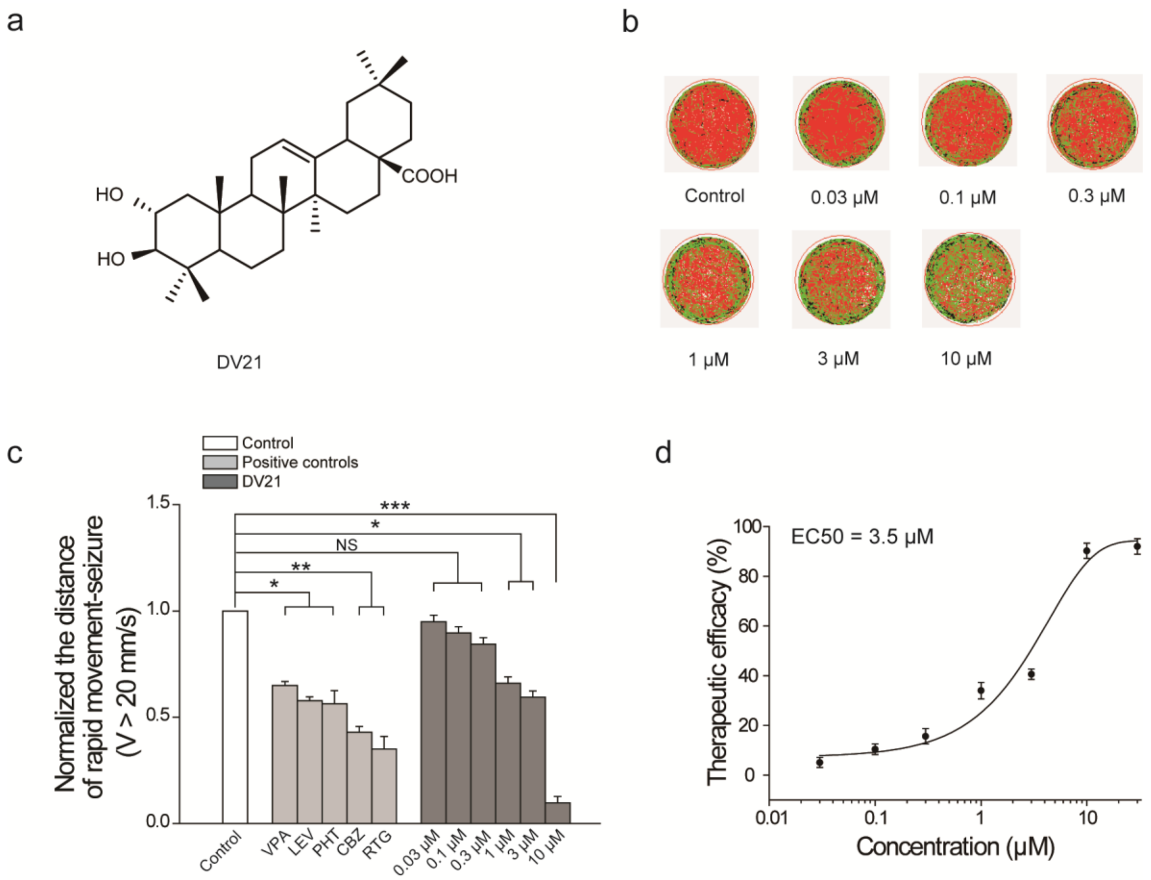

Supplementary Fig. 1. Screening for natural compounds with anti-seizure activity. (**a**) The spatial structure of DV21. (**b**) The trajectories of zebrafish epilepsy model under different concentrations of DV21. (**c**) Compare the effects between different concentrations of DV21 and clinical drugs. Two-way ANOVA was used. (**d**) The EC_{50} of DV21 on the zebrafish epilepsy model. ***$P < 0.001$; **$P < 0.01$; *$P < 0.05$. Error bars are means ± s.e.m. NS, not detectable.

VPA: sodium valproate; LEV: levetiracetam; PHT: Phenytoniumnatricum; CBZ: carbamazepine; RTG: retigabine.

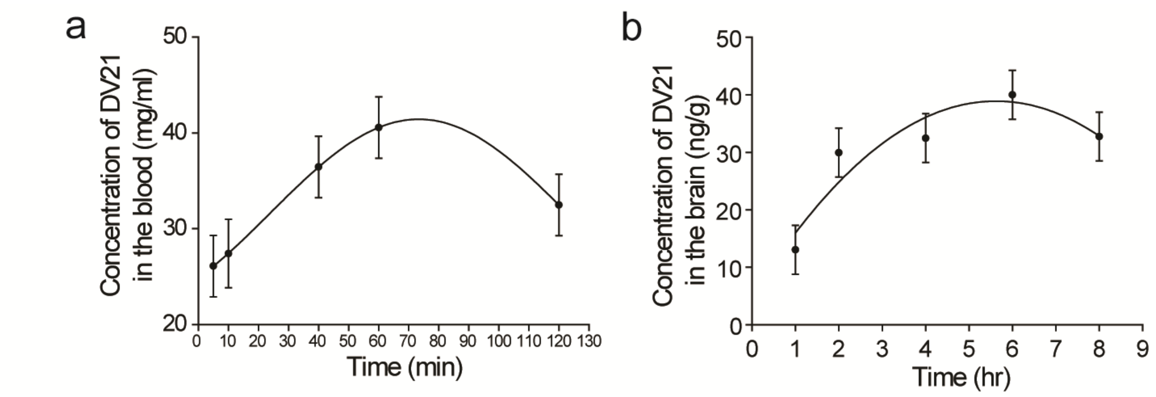

Supplementary Fig.2. The concentrations of DV21 in the blood and brain. (**a**) Concentrations of DV21 in the blood (n = 5). (**b**) Concentrations of DV21 in the brain (n = 5).

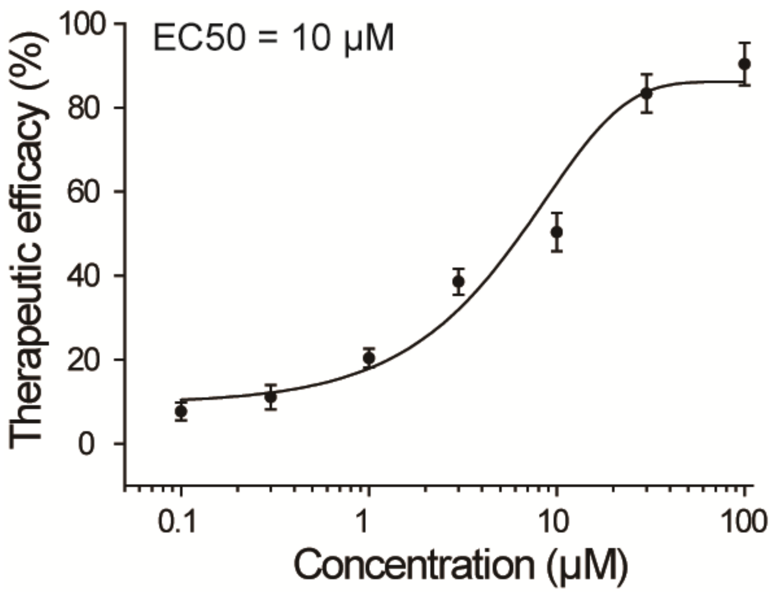

Supplementary Fig. 3. The concentrations of EC50 of DV21 on cortical pyramidal neurons (n = 14).

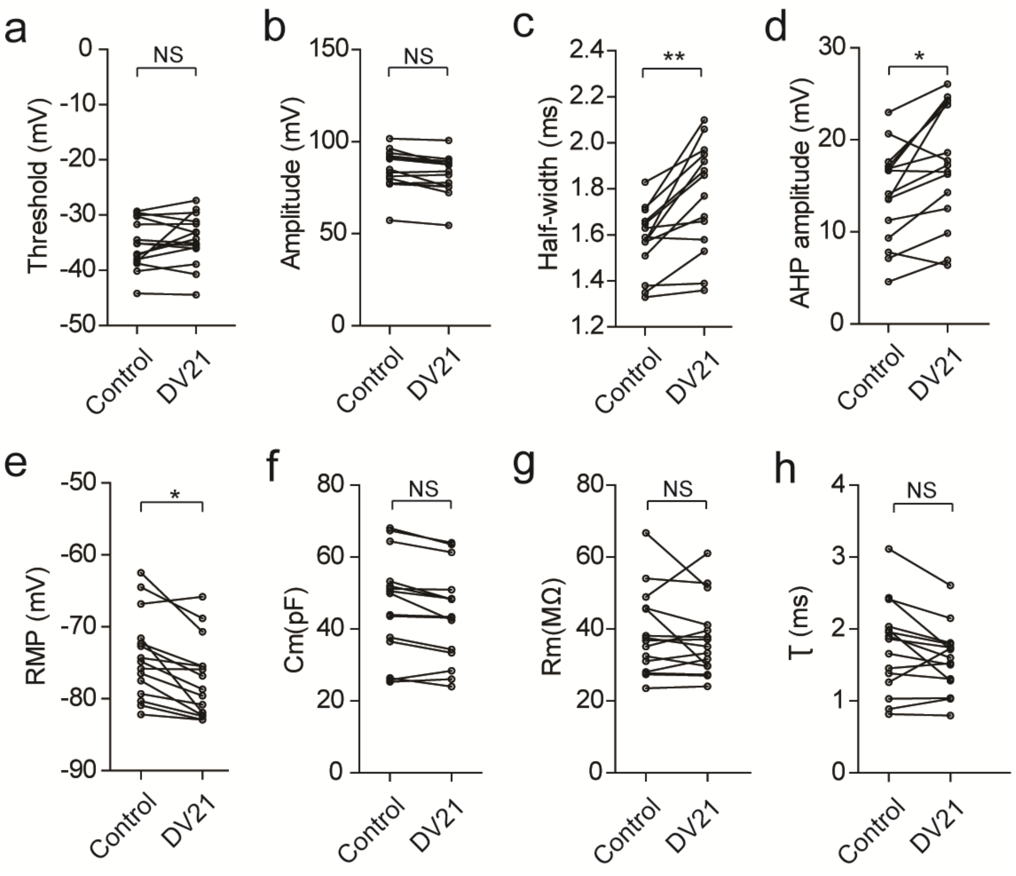

Supplementary Fig. 4. Electrophysiology character of cortical pyramidal neurons. (**a, b**) Effects of DV21 on threshold and amplitude of cortical pyramidal neurons. (**c, d**) Effects of DV21 on the half-width and AHP amplitude of cortical pyramidal neurons. (**e**) The effect of DV21 on the resting membrane potential (RMP). (**f, g, h**) Effects of DV21 on the membrane resistance (Rm), membrane capacitance (Cm) and membrane time constant (τ_m). n = 15, ***P*< 0.01; **P*< 0.05. *P*-value was calculated by two-sided *t*-test. Error bars are means ± s.e.m. NS, not detectable.

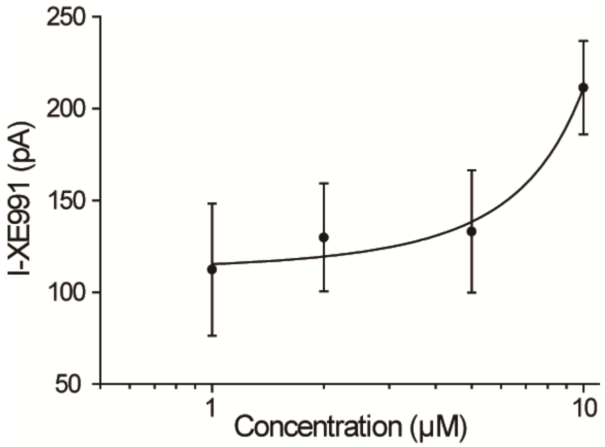

Supplementary Fig. 5. The amplitudes of M current on different concentrations of DV21, as the concentration of DV21increases, the amplitude of M current increased. (0 μM: n = 11; 1 μM: n = 6; 2 μM: n = 9; 5 μM: n = 7; 10 μM: n = 12).

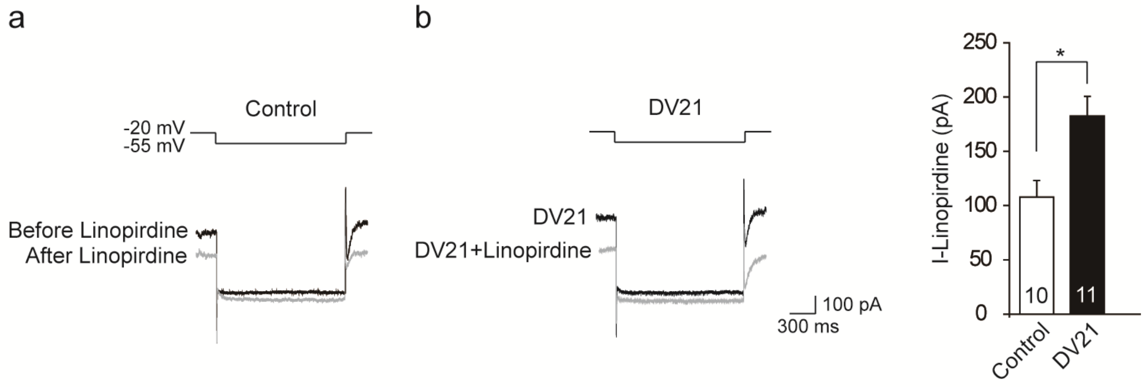

Supplementary Fig. 6. Contrasting the effects of DV21 on the M current after partly block the M-channels by linopirdine. The M current protocol and representative M current traces. M current were induced by a step hyperpolarization (1.5 s) to -55 mV from a holding potential of -20 mV in pyramidal neurons from control (**a**) and DV21 (**b**) groups before and after the application of 10 μM linopirdine. (**c**) Summary histogram showing the effect of DV21 on M current. (n = 10, control; n = 11, DV21.) *P*-value was calculated by two-sided *t*-test. **P* < 0.05. Error bars are means ±s.e.m.

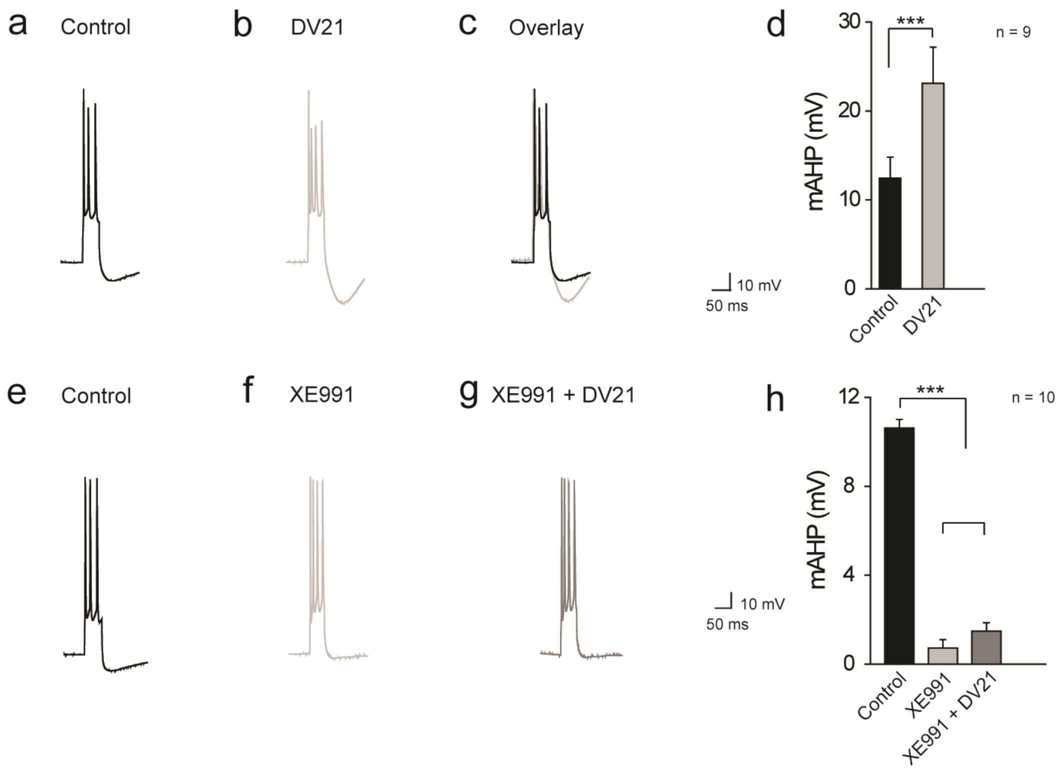

Supplementary Fig.7. Comparison of medium afterhyperpolarizations (mAHPs) following spike trains in cortical pyramidal neurons. Representative recordings of the mAHPs recorded in normal medium (**a, e**) and after application of DV21 (**b**), XE991 (**f**), XE991+DV21 (**g**). Traces are shown superimposed (**c**), mAHP increased after administration of DV21. Summary diagrams compare mAHPs peak amplitudes following one to four action potentials before and after application of DV21(**d;** n = 9), application of XE991 significantly reduced mAHPs and then DV21 had little or no effect (**h;** n = 10). ***P < 0.001.Error bars are means ±s.e.m.

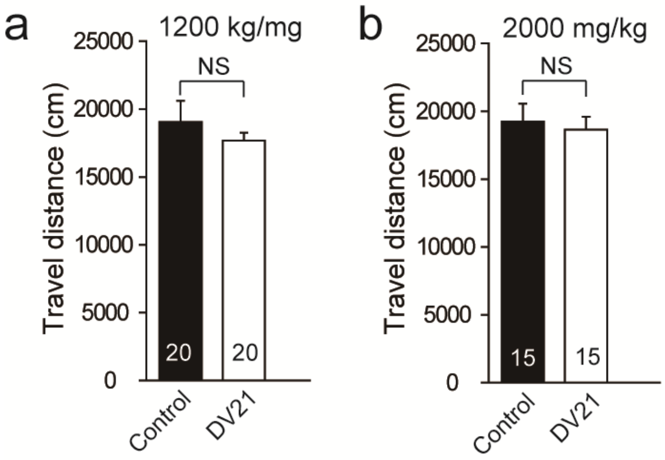

Supplementary Fig.8. Travel distances in the open field within 30 minutes. (a). Summary histogram showing the effect of DV21 with 1200mg/kg on mobility. n = 20. (b). Summary histogram showing the effect of DV21 with 2000mg/kg on mobility. n = 15. *P*-value was calculated by two-sided *t*-test.Error bars are means ± s.e.m. NS, not detectable.
